# The Effects of *Ficus carica* on Male and Female Reproductive Capabilities in Rats

**DOI:** 10.1155/2022/1799431

**Published:** 2022-10-22

**Authors:** Noor ul ain, Rafeeq Alam Khan, Talat Mirza, Talha Bin Fayyaz

**Affiliations:** ^1^Department of Pharmacology, University of Karachi, Karachi, Pakistan; ^2^Department of Pharmacology, Institute of Pharmaceutical Sciences, Jinnah Sindh Medical University, Karachi, Pakistan; ^3^Faculty of Pharmacy, Ziauddin University, Education City Link Road Karachi, Karachi, Pakistan; ^4^Medical College, Ziauddin University, Karachi, Pakistan

## Abstract

The basic purpose of pharmacology is to look into the benefits of natural remedies and make them available to the general populace. Herbal medicines are now considered to be the future of humanity. The current study explored the effects of *Ficus carica* (FC) extract in rats of two-generation F_0_ (parents) and F_1_ (offspring) in either sex. The *F. carica* extract was initially tested for acute and sub-chronic toxicity. Extracts were also tested for fertility assessment and effects on reproductive hormones like follicle-stimulating hormone (FSH), luteinizing hormone (LH), gonadotropin-releasing hormone (GnRH), estradiol, testosterone, dihydrotestosterone (DHT), dehydroepiandrosterone-sulfate (DHEAS), insulin, progesterone, and prolactin. The antioxidant activity of the extract was also evaluated by testing mRNA expressions of SOD2, GPX1, CAT, and GR in male testes and female ovaries. The animals treated with 100 mg/kg FC extract produced a more pronounced fertility effect in both genders. Expression of CAT, SOD2, GPX1, and GR was found to be increased in the reproductive organs of both sexes. Histology of the testes reveals increased spermatogenesis and increased folliculogenesis in ovaries. The hormone profile showed an increase in FSH, DHT, estradiol, and DHEAS levels in males and FSH, LH, estrogen, and DHEAS in females. The results of the study establish the effectiveness of natural products in improving fertility issues in either sex.

## 1. Introduction

*Ficus carica Linn*. (figs) had been traditionally used as a laxative and for other medicinal benefits related to cardiovascular, respiratory, spasmolytic, and anti-inflammatory effects [1]. The leaves of *F. carica* (*Moraceae*) have been assessed for hepatoprotective effect in carbon tetrachloride-induced liver toxicity in rats [2]. The therapeutic benefit of plants is often attributed to their antioxidant properties [3]. The use of this fruit may prevent homeostatic diseases caused by oxidative features [4]. It seems that their usage supports the prevention of vein blockage; its high fiber content has a laxative effect, and its latex deters the growth of carcinoma cells [5]. *F. carica* contains three hydroxycinnamic acids (3-O- and 5-O-caffeoylquinic acids and ferulic acid), two flavonoid glycosides (quercetin 3-O-glucoside and quercetin 3-O-rutinoside), and two furanocoumarins (psoralen and bergapten). Except for ferulic acid, all other phenolic compounds are present in the leaf, pulp, and peel [4]. *F. carica* is rich in amino acids including five essential amino acids histidine, leucine, lysine, phenylalanine, and tryptophan, and eight nonessential amino acids like alanine, and asparagine, cysteine, glutamine, glycine, ornithine, serine, and tyrosine. Cysteine, tryptophan, and tyrosine are found in greater concentrations than other amino acids [4, 6]. *F. carica* also contains coumarin which possesses anticancer, antianemic, and antioxidant activities. It has an alkaloid (quinine) that possesses antimalarial activity, anthocyanin (cyanidin-3-O-glucoside, cyanidin-3-O-rhamnose glucoside) with antioxidant and radical scavenging actions, as well as hydrocarbon which possesses antioxidant, hemoptysis, and antiseptic activities [6]. The objective of the current study was to determine the effect of long-term exposure to *F. carica* on the liver, kidney, hematological parameters, and fertility in male and female rats.

## 2. Materials and Methods

The research was executed after the consent from the Board for Advanced Studies and Research, the University of Karachi, allocated number 04642/pharm, dated September 2019, and followed by the permission of the DRC, Department of Pharmacology to use the animals as per the guidelines of the NIH.

### 2.1. Extract Preparation

*F. carica* (FC) fruits were procured from a fruit shop in Karachi; Prof. Huma Shareef at the Department of Pharmacognosy Institute of Pharmaceutical Sciences, JSMU, Karachi identified it. The 70% hydro ethanol extract of FC was prepared as per the method of Elghareeb [7], the ripened fruits were sliced into small fragments and kept to dry at 40°C in a hot oven then ground with the help of a grinder. The fine powdered material was assorted with 70% aqueous ethanol (1 : 5 weight/volume) for about 72 hours, along with continuous shaking. The soaked material was cleansed from plant fragments with a fine filter paper and vaporized under condensed pressure on a rotatory evaporator. The extract was further dried, lyophilized, and kept at−20°C till further use. The yield percentage of *F. carica* extract was 25% (w/w) [7].

### 2.2. Animals

The albino Wistar rat used in the study were aged 11–13 weeks (11 weeks for male and 13 weeks for females) and raised in the animal's house of ICCBS, University of Karachi. Animals weigh about 170 to 200 grams. The rats were housed in a room maintained at 25°C; with 45% humidity and exposed to a natural daily 12 hr light/dark cycle. The rats were retained in the standard cages in the animal house. Pelleted rodent food and filtered tap water was provided ad libitum. The rats were acclimatized for two weeks. Females were subjected to vaginal smear examination for two weeks before treatment. The vaginal samples were collected by separating the lips of the female genital organ and placing them in a sterile cotton swab into the vagina. The swab was turned two to three times against the vaginal wall to collect the smear and moved on a clean glass slide; the smears were air-dried and stained with Wright Giemsa stain and observed by the microscope (×100 magnification) [8]. Females showing regular 4-day estrous cycles were used in the present study. Three days before the start of testing female rats were exposed to the filthy bedding of a male rat to harmonize their estrous cycles [9]. Vaginal smears were then examined daily, and on the first day of pregnancy were identified in the morning when spermatozoa were found.

### 2.3. Acute Toxicity

The acute toxicity study was conducted following OECD Guideline-423 a healthy albino Wistar female rat with 170–200 gm weight was used in the present study. The experiment was conducted on three female rats. Under fasting conditions, the rats were examined following the administration of a single dose of 500 mg/kg FC extract; food was suspended for another 4 hours after administration of the extract. Toxicity markers were noted for the first 24 hours, every hour, and each day for 14 days from the start of the study. The rats were under surveillance for 14 days and observed body weight, posture, signs of seizure, limb paralysis, body tone, gross behavioral changes, and mortality [10].

### 2.4. Sub-Chronic Toxicity

Wistar rats in three groups of ten animals (five males and five females) were given 70% FC hydroethanol extract by mouth in doses of 50 mg/kg and 100 mg/kg as a single daily dose for 90 days. The control animals were given the vehicle. All rats were kept in the same environments, with food and water available at all times, throughout the study. Toxicity was evaluated through gross behavior, blood chemistry, and enzyme analysis. The plasma was obtained by collecting blood in EDTA tubes which were then used for blood analysis chemistry and enzyme analysis (AST, ALT, alkaline phosphatase, bilirubin, troponin I&T, CKMB, BUN, and creatinine).

### 2.5. Fertility Assessment

Virgin female albino Wistar rats aged 13 weeks and male albino Wistar rats aged 11 weeks were divided into 3 groups. Each group consists of six males and six females. Dosing was started 70 days before the mating and continued throughout the mating in males and continued in females throughout gestation and lactation. Three days before mating, the female rats were exposed to the soiled bedding of a mature male rat to synchronize their estrous cycles [9]. Day-1 pregnancy was marked after the formation of a vaginal plug. Male and female rats were paired up for breeding, and after 14 days, rats were removed from the female cages for the safety of pups.

The exact doses of the FC extract were given to certain F_1_ generation offspring when weaned and throughout their growth and maturation to adulthood, as well as during the breeding. Females of the F_1_ generation who were pregnant continue to get the FC extract throughout their pregnancy until the F2 generation offspring were weaned. From each litter, two males and two females were chosen at random; for mating with another pup of a different litter at the same dose, and level to produce the next generation. One-week acclimatization period was given before the exposure to FC extract to the F_1_ generation. The groups were formed in a pattern similar to the F_0_ generation and mated after 12 weeks of maturity, males received dosing throughout mating, and females received dosing throughout the gestational and lactation period. Each litter was examined immediately on delivery for the number of pups, stillbirths, live births, and the presence of overall defects. The neonates were cautiously witnessed on postnatal days zero (birth), four, seven, fourteen, and 21 days. Dead pups were grossly scrutinized for probable defects and the cause of disease. Reproductive toxicity endpoints show the animal's retorts to the FC extract from conception to weaning. This analysis reflects the total effect of the FC extracts at all stages, which is a more profound indicator for reproduction studies. Animals of the F_0_ and F_1_ generations were divided into three groups as follows:

- The control group comprised six males and six females given once daily 1 ml of distilled water by mouth.
- Treated groups were given 50 mg/kg and 100 mg/kg of FC extract by gastric gavage tube.

### 2.6. Blood Assessment

Blood samples for hormonal measurement were obtained during the estrus phase. The cardiac puncture procedure was used to collect blood samples. The blood was allowed to stand for 10 minutes in centrifuge tubes to clot at area temperature and centrifuged at 3500 rpm for 10 minutes. The serum was transferred to a different container, and the sera were stored at−80°C. ELISA was used for the assessment of testosterone, LH, FSH, estrogen, progesterone, prolactin, DHT, GnRH, DHEAS, and Insulin.

### 2.7. Histological Procedures

Ovaries of the control group were removed during the estrus phase. Excised ovaries were protected in 10% formalin after cleaning the adherent connective tissues, then processed by usual procedures and embedded in paraffin. The ovaries were longitudinally and serially sectioned at five *μ*m thickness, and the mounted sections on a glass slide were deparaffinized by xylene. Ethanol series (100, 90, 80, 70, and 50%) and distilled water were used for rehydration and stained using Hematoxylin and Eosin. Stained microscopic slides were evaluated and compared in groups. The numbers of preantral, antral, corpus luteum, corpus Albicans and cystic follicles, and the stromal reaction were determined [11].

The testes of the selected rats from all the groups in the study were detached and weighed. The testes were fixed in Bouin's fluid at room temperature for 24 h, tan tissues were handled routinely. For a short, while the tissues were transferred to 70% alcohol and dried by moving through ascending grades of alcohol; the tissues were then cleared in xylene and lastly embedded in paraffin wax 5 m sections were cut using a rotary microtome and stained with hematoxylin and eosin. The stained slides were seen in the microscope and images were taken.

### 2.8. Polymerase Chain Reaction (PCR)

RNA from the ovaries and testes was extracted by the triazole method. Reverse Aid First-strand complementary DNA (cDNA) synthesis kit (Thermo Scientific) was used for the synthesis of first-strand cDNA from 1 *μ*g of total RNA. The oxidative stress enzyme indicators, i.e., CAT, SOD2, GPX1, and GR expressions in ovaries and testes were evaluated by RT-qPCR. The total reaction mixture volume was 25 microliters with thermal cycling in three steps. The reaction mixture was first gone through denaturation for 10 min at 95°C then again denatured for 15sec at 95°C followed by annealing for 30 sec at 60°C and final extension for 30 sec at 72°C. Denaturation, annealing, and extension comprised 40 cycles. Thermo Scientific Maxima SYBR Green/ROX qPCR master mix (2X) kit was used and the parallel sense and antisense were shown in Table 1. Primers were acquired from The Worldwide Scientific. The difference in gene expression between the control and treated groups was reported by the delta-delta cycle threshold (Ct) method [12]. GAPDH was used as a reference housekeeping gene. Primer sequence (5′⟶3′) of GAPDH, SOD2, CAT, GPX1, and GR [13].

### 2.9. Statistical Analysis

Data were stored and analyzed using IBM-SPSS version 23.0. Mean with standard error to the mean was reported for liver, cardiac, kidney markers, blood profiles, and F_0_ and F_1_ generations hormone profiles in each studied group. These means were matched by one-way ANOVA, and post hoc analysis was done using Tukey's test for multiple comparisons. *P* < 0.05 was deliberated statistically significant, and *P* < 0.01 was highly significant. Data were also compared for male and female samples separately in F_0_ and F_1_ generation parameters.

## 3. Results

### 3.1. Acute Toxicity

All animals were given the *F. carica* extract in the dose of 500 mg/kg survived during 14 days of observation. Oral administration of this extract at 500 mg/kg did not yield any substantial changes in behavior, breathing, skin effects, defecation, loss of hair, postural abnormalities, lacrimation, salivation, loss in food intake, reflex sensitivity, and body weight of female rats.

### 3.2. Sub Chronic Toxicity

The *F. carica* extract at 50 and 100 mg/kg exerted no side effects during the 90 days of the treatment period. However, there was an increase in animal body weight as compared to the control after 90 days. The percentage weight change in the males treated with 50 mg/kg was 28–34% and those treated with 100 mg/kg was 31–42%, while in females the percentage weight change at the same doses was 20–30%, and 25–35%, respectively, as compared to control.

Table 2 summarizes the data regarding the effect of FC extract on aspartate aminotransferase (AST), alanine transaminase (ALT), total bilirubin, and alkaline phosphatase (ALP), troponin I, troponin T, creatine kinase-MB (CKMB), and creatinine and blood urea nitrogen (BUN). A significant decrease in ALT and total bilirubin at both doses was observed as compared to the control. There was a significant decrease in troponin I at 50 mg/kg and 100 mg/kg and a significant decrease in the levels of troponin T at 100 mg/kg as compared to the control.

Table 3 shows hematological parameters; there was a significant increase in RBC and platelets count in the groups treated with 50 mg/kg and 100 mg/kg as compared to the control.

### 3.3. Fertility Evaluation

Tables4 and 5 reveal the pharmacological evaluation of various fertility parameters in F_0_ and F_1_ generation of albino Wistar rats following the administration of the two doses against a control group. The F_0_ generation (Table 4) treated with FC extract 50 mg/kg showed approximately a 17% increase in total pregnancies, whereas FC 100 mg showed a 33% increase in total pregnancies compared to the control. In animals given FC 50 mg/kg conception occurred in 15 days while in animals receiving FC100 mg/kg conception occurred in 3.2 days in comparison to control in which conception occurred in 27.3 days. The gestational period was decreased in both treated groups to 20 days as compared to 21 days in the control group.

There was an increase in the mean number of pups/litter in animal groups treated with 50 and 100 mg/kg of the FC extract, i.e., eight litter sizes compared to 7.3 litter sizes in control. The mean weight of pups at birth was increased to 27 and 32 g in animals treated with 50 and 100 mg/kg of the FC extract, respectively, as compared to 21 days of control in animals. The male-to-female ratio increased in FC50 and FC100 mg/kg as compared to the control.

The F_1_ generation (Table 5) treated with FC extract 50 mg/kg showed approximately a 17% increase in total pregnancies, whereas FC 100 mg showed a 50% increase in total pregnancies as compared to the control. In animals that were given FC 50 mg/kg, conception occurred in 7.6 days while animals that received FC100 mg/kg, conception occurred in 1.6 days in comparison to control where conception occurred in 27.3 days. The gestational period was decreased in both treated groups to 20 days as compared to 21 days in the control group.

There was an increase in the mean number of pups/litter in animal groups treated with 50 and 100 mg/kg of the FC extract, i.e., eight litter sizes compared to 7.5 litter sizes in control. The mean weight of pups at birth was increased to 29 and 35 g in animals treated with 50 and 100 mg/kg of the FC extract respectively as compared to 24 gram of control animals. The male-to-female ratio increased in FC50 mg and FC 100 mg was also increased as compared to the control.

### 3.4. Hormone Profile of F_0_ and F_1_ Generation

Table 6 displays a comparison of hormone levels in both sexes of control animals and F_0_ generation treated with two different doses of *F. carica*. There was a significant increase in GnRH levels in adult males treated with FC 100 mg/kg as compared to control rats, while there was also a significant increase in DHEAS at the same dose of FC. *F. carica* extract at both doses revealed a highly significant increase in the levels of FSH and estradiol in the rats of either sex of F_0_ generation as compared to control. There was also a highly significant increase in the levels of LH in male rats treated with both doses of FC, whereas LH was only significantly increased in female rats at 50 mg of FC extract. The DHT levels were increased highly significantly only in male rats treated with both doses of FC extract as compared to the control. The level of DHEAS was found to increase highly significantly in the male rats treated with FC at 50 mg and100 mg/kg, whereas in female rats it only increased significantly at 100 mg/kg. The levels of insulin were increased significantly at 50 mg/kg and a highly significant increase was observed at 100 mg/kg in male rats, while the levels of prolactin in female rats at 100 mg/kg also showed a highly significant increase as compared to control.

Table 7 displays a comparison of hormone levels in both sexes of control and F_1_ generation animals treated with two different doses of *F. carica*. There was a highly significant increase in the levels of FSH in adult males treated with FC at both doses, 50 and 100 mg/kg; however, in female rats, there was a highly significant increase only in FSH levels at 100 mg/kg as compared to control rats. There was a highly significant increase in the levels of LH in both male and female rats treated with 50 and 100 mg/kg of FC extract, while estradiol was increased highly significantly in female rats treated with 50 and 100 mg/kg of FC extract.

The levels of DHEAS in both genders of the F_1_ generation were increased highly significantly at 50 and 100 mg/kg FC extract as compared to the control.

### 3.5. Microscopic Examination

#### 3.5.1. Histology of Ovaries

**Figure 1:**
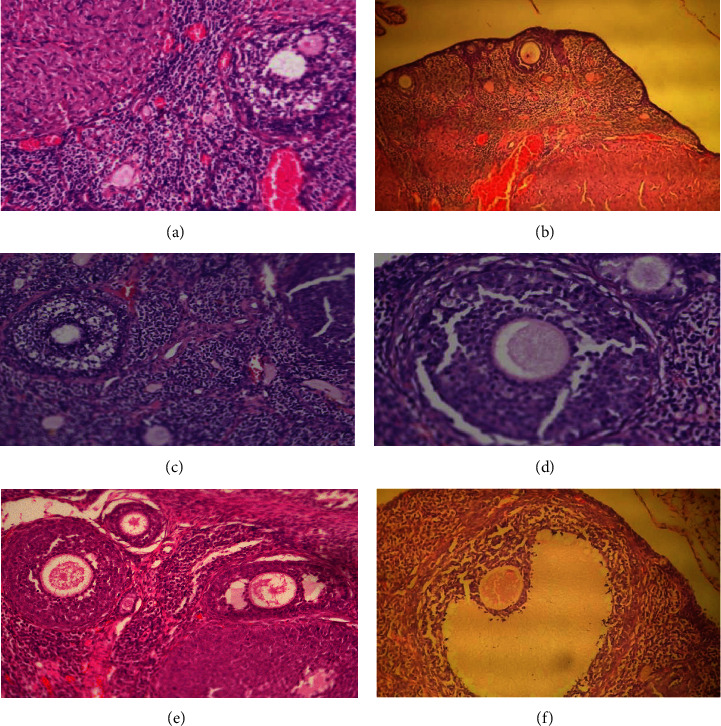
(a) Micrograph of control rat ovary F_0_ generation showing secondary antral follicles 20x. (b) Micrograph, control rat ovary F_1_ generation showing primary follicle in the cortex 10x. (c) Micrograph, FC 50 mg treated rat ovary F_0_ generation showing primary follicle 20x. (d) Micrograph, FC 100 mg treated rat ovary F_0_ generation showing primary follicle 40x. (e) Micrograph, FC 50 mg treated rat ovary F_1_ generation primary follicle 40x. (f) Micrograph, FC 100 mg treated rat ovary F_1_ generation showing mature griffin follicle 40x.

The microscopic assessment of ovarian slices of the control group in F_0_ and F_1_ generation reveals that the ovaries consist of the external cortex protected with an intact capsule with abundant stroma. The ovarian exterior epithelium forms a single layer of flattened cells and is separated from the underlying tunica albuginea (Figures 1(a) and 1(b)). In the medulla, many blood vessels separated by unattached connective tissue and interstitial glandular cells are distributed (Figure 1(a)).Folliculogenesis was observed. Follicles in different stages of development were present, ranging from primary, secondary, and corpora lutea. At high magnification, primordial follicles, the primary follicle of an intact primary oocyte surrounded by a single layer of cuboidal epithelium placed beneath the tunica albuginea can be seen. The stratified epithelium of granulosa cells surrounds the secondary follicle. Histological observations of the ovary of the control group showed typical follicular growth along with corpora lutea, primary follicles, and secondary follicles. Folliculogenesis is observed in all the samples.

Whereas, microscopic checkup of ovarian slices of the F_0_ (Figures 1(c) & 1(d)) F_1_ female generation (Figures 1(e) & 1(f)) treated with FC group reveals that the ovary contained the thin outer layer of the cortex covered with an intact capsule with abundant stroma. The ovarian exterior epithelium is made of a single layer of flattened cells and is separated from the underlying tunica albuginea (Figures 1(c)–1(f)). In the medulla, several blood vessels and interstitial glandular cells were distributed (Figure 1). Folliculogenesis can be observed but more prominent in the groups treated with FC 100 mg/kg (Figures 1(d) and 1(f)) of F_0_ and F_1_ generation. Follicles in different stages of development were present, ranging from primary, secondary, tertiary follicle, and corpora lutea. At high magnification, primordial follicles, the primary follicle of an intact primary oocyte surrounded by a single layer of cuboidal epithelium placed beneath the tunica albuginea can be seen. The secondary follicle is surrounded by the stratified epithelium of granulosa cells (Figures 1(c)–1(e)). A tertiary follicle or mature griffin follicle with an intact antral cavity was present (Figure 1(f)). Histological observations of the ovary of the control group showed typical follicular growth along with corpora lutea, primary follicles, secondary follicles, and tertiary follicles. Folliculogenesis is observed in all the samples. The histological analysis of the experiment groups is summarized in Table 8.

#### 3.5.2. Histology of Testes

**Figure 2:**
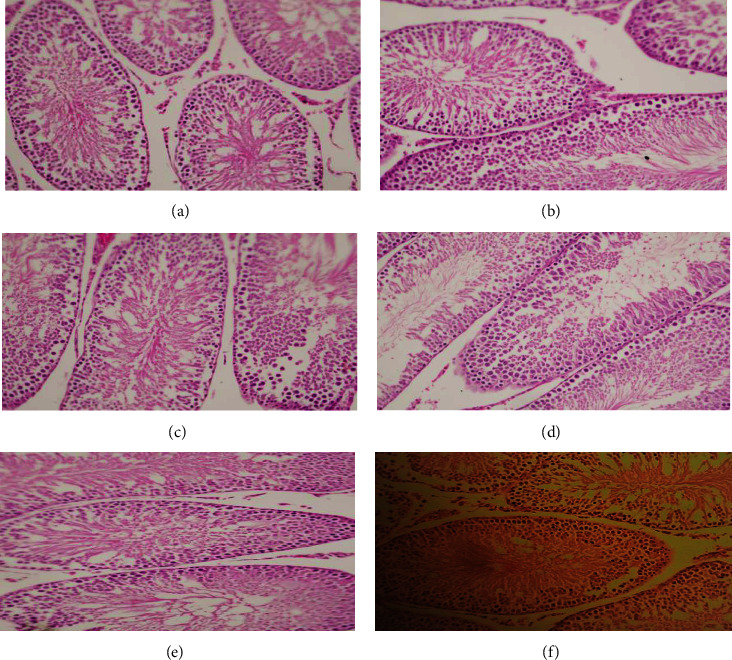
(a) Micrograph of the control rat testes F_0_ generation showing seminiferous tubules 20x. (b) Micrograph of the control rat testes F_1_ generation showing seminiferous tubule 20x. (c) Micrograph of the F_0_ generation male testes treated with 50 mg/kg *FC* extract 20x. (d) Micrograph of the F_0_ generation male testes treated with 100 mg/kg *FC* extract 20x. (e) Micrograph of the F_1_ generation male testes treated with 50 mg/kg FC extract 20x. (f) Micrograph of the F_1_ generation male testes treated with 100 mg/kg FC extract 20x.

The microscopic examination of the testes of the control group of F_0_ and F_1_ Figures 2(a) and 2(b) shows the normal architecture of seminiferous epithelia of tubules with spermatogenic cells and Sertoli cells in the base of the tubule and interstitial area with minimal connective tissue surrounding the tubules. The microscopic examination of the testes of all the treated groups of F_0_ and F_1_ generation shows the normal architecture of seminiferous epithelia of tubules with spermatogenic cells and Sertoli cells in the base of the tubule and interstitial area with minimal connective tissue surrounding the tubules. However, closer to the tubule's core, there are primary and secondary spermatids and sperms in the center of the lumen of the seminiferous tubule. Increased production of sperms was observed in the groups treated with 100 mg/kg FC in F_0_ and F_1_ generation compared to the respective controls. Johnsen's score of F_0_ generation rat treated with FC50 samples was 9.66 ± 0.52 and of FC 100 samples was 10 ± 0 as compared to the control with a score of 9.66 ± 0.52. Whereas in F_1_ Johnsen's score of FC50 samples was 9.6 ± 0.52 and of FC100 samples was 9.83 ± 0.41as compared to control with a score of 9.5 ± 0.55. The histological samples of the testes did not reveal any abnormality like seminiferous tubular distortion or hyperplasia in the Leydig cell.

### 3.6. Antioxidant Effects of FC on F_0_ Generation

**Figure 3:**
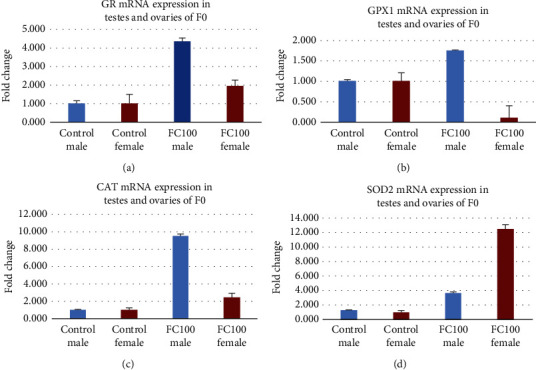
(a): Effect of FC the extract on glutathione reductase. (b) Effect of the FC extract on glutathione peroxidase. (c) Effect of the FC extract on catalase. (d) Effect of the FC extract on superoxide dismutase.

Figure 3 reveals the effect on gene expression of glutathione reductase (GR), glutathione peroxidase (GPX1), catalase (CAT), and superoxide dismutase (SOD2) in the group treated with *F. carica* at 100 mg/kg. Gene expression analysis of these enzymes by RT-qPCR shows that all enzymes were upregulated in the ovarian and testicular homogenates of all treated groups in the F_0_ generation as compared to the control of the F_0_ generation; however, GPX1 mRNA expression in the female of F_0_ generation at 100 mg/kg was downregulated.

### 3.7. Antioxidant Effects of FC on the F_1_ Generation

**Figure 4:**
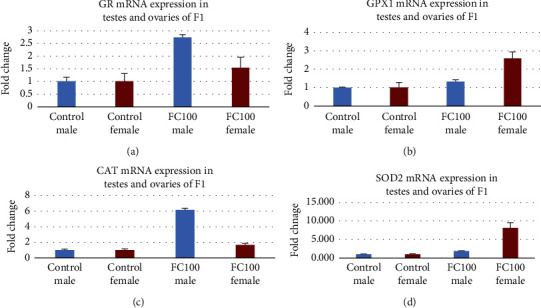
(a) Effect of the FC extract on glutathione reductase. (b) Effect of the FC extract on glutathione peroxidase. (c) Effect of the FC extract on catalase. (d) Effect of the FC extract on superoxide dismutase.

Figure 4 reveals the effect on gene expression of glutathione reductase (GR), glutathione peroxidase (GPX1), catalase (CAT), and superoxide dismutase (SOD2) in the group treated with FC at 100 mg/kg. Gene expression analysis of these enzymes by RT-qPCR shows that all enzymes were upregulated in the ovarian and testicular homogenates of all treated groups in the F_1_ generation as compared to the control of the F_1_ generation.

## 4. Discussion

The results of the acute toxicity study indicate that 70% hydro ethanol extract of *F. carica* fruit is safe up to 500 mg/kg, and none of the animals died during 14 days of the administration, while sub-chronic toxicity data indicate that *F. carica* fruit extract possesses protective effects on the liver as it decreased ALT and total bilirubin levels [14]. The results of the present study are in line with the previous study showing the hepatoprotective effect of *F. carica* fruit against gamma-radiation-induced hepatotoxicity [14]. *F. carica* caused a significant decrease in the concentration of Troponin I and troponin T at 100 mg/kg as compared to the control. Decreased levels of troponin show the cardio-protective effect of *F. carica* extract, while the renal profile was comparable to the control. Thus, results of the current study show the overall cardiac and renal protective effect of *F. carica* supported by previous studies which reveal FC extract to have potent antioxidants and protective effects against cardiac and renal toxicity induced by 5-FU [7]. Hematological studies reveal anomalies in body metabolic processes and furnish vital information on the body's response to injury, deprivation, and stress [15]. *F. carica* at 100 mg/kg caused a highly significant increase in RBC count while a highly significant increase in platelet counts in both the groups treated with 50 mg/kg and 100 mg/kg as compared to the control. Thus, we may conclude that hydro ethanol extract of *F. carica* may be effective in the treatment of dengue.

One of the goals of this study was to evaluate the parameters related to male and female fertility and its effects on two-generation following exposure to *F. carica* extract during gestation. *F. carica* increased fertility in a dose-dependent manner, i.e., the group treated with 100 mg/kg showed better results in F_0_ and F_1_ generation than the group treated with 50 mg/kg. It increased the fertility index; the mean number of pups/litter and copulation was less than the control. There was an increase in the pup's weight at the time of birth and throughout 21 days, while pups showed increased growth as compared to the pups in the control group.

*F. carica* at 50 mg/kg revealed a highly significant increase in the levels of FSH, LH, estradiol, DHT, and DHEAS in males of the F_0_ generation as compared to control, while insulin was increased significantly. Whereas, in the group treated with 100 mg/kg of *F. carica,* there was a highly significant increase in the levels of FSH, LH, estradiol, DHT, DHEAS, and insulin, moreover the levels of GnRH were also increased significantly, while in the males of F_1_ generation treated with *F. carica* 50 and 100 mg/kg, the levels of FSH, LH and DHEAS were increased highly significant as compared to control.

Both FSH and LH enhance testicular growth in males while estradiol, DHT, and DHEAS are essential for libido, erectile function, and spermatogenesis in males [16]. The findings of this study are in line with the study by Allan et al. [17] who revealed that testosterone and FSH are essential for the optimal regulation of spermatogenesis individually or synergistically. Moreover, spermatogenesis can be restored in hypo-gonadal mice by the administration of testosterone or its metabolite, dihydrotestosterone [17]. The levels of progesterone and testosterone in animals treated with 50 and 100 mg/kg *F. carica* extract were almost comparable to the control. There has been an overall increase in gene expression of antioxidant enzymes GR, GPX1, SOD2, and CAT. Estradiol enhanced glutathione peroxidase (GPX) in the liver [18]. Hence we may conclude that in the present study the increase in expression of GPX1 in testes may also be due to an increase in estradiol levels in male rats by *F. carica*. Whereas, in the males of the F_1_ generation, a highly significant increase in the levels of FSH, LH, and DHEAS was observed in a dose-dependent manner. GnRH, estradiol, DHT, testosterone, progesterone, and insulin were comparable to control animals. *F. carica* increased the levels of FSH more than that of LH, DHT, and estrogen which is therefore responsible for the increase in fertility in parent males. The outcomes of the present study are in accordance with a previous study by Haredy et al. [19], who reported a reduction in lithium carbonate-induced liver toxicity by *F. carica* and increased endogenous activity of antioxidant enzymes (CAT and SOD2) and semen quality, while there was a significant decrease in markers of oxidative stress in testes, i.e., NO, 8OHdG, MDA and GSSG. Thus, it may be concluded that increased gene expression tends to reduce oxidative stress and in turn increases overall reproductive activity.

Animals treated with 100 mg/kg FC showed increased expression of antioxidants in the testes with an increase in the conversion of testosterone into estrogen and DHT, while there was also an increase in FSH levels, Abu Bakar et al. [20] showed that *F. carica* increases testosterone production and has a positive effect on FSH. This indicates that *F. carica* has the potential to increase spermatogenesis in males which was also evident by histological changes in testes as well as fertility data in both generations showing conception and copulation more efficiently without any toxicity in the groups treated with *F. carica* in a dose-dependent manner.

The previous study shows that in insulin-dependent diabetes testosterone synthesis and function of Leydig cells are decreased due to the lack of insulin, thus reducing LH levels, sperm output, fertility, and FSH [21]. However, in the present study, *F. carica* has significantly increased insulin in males of the F_0_ generation and may be responsible for increased fertility due to elevated levels of FSH and LH.

In females of the F_0_ generation, there was a highly significant increase in the levels of FSH and estradiol, while LH was increased significantly in both groups treated with *F. carica*, whereas there was a significant increase in DHEAS level at 100 mg/kg and a highly significant increase in prolactin levels. In contrast, females of the F_1_ generation showed a highly significant increase in the levels of FSH at 100 mg/kg and a highly significant increase in estradiol and DHEAS in both the groups treated with *F. carica* in a dose-dependent manner.

FSH is crucial for the survival, propagation, and distinction of preovulatory mural granulosa and excites the expression of hundreds of genes, comprising those required for the production of estradiol and expression of the LH receptor [22]. The high level of estradiol formed by the emerging principal follicle employs positive feedback on the anterior pituitary resulting in a surge of FSH and LH into the circulation. This surge in LH acts on mural granulosa cells, and yields high levels of the LH receptor, to initiate a series of events leading to cumulus cell expansion and follicle rupture. The treatment with *F. carica* increased the level of FSH, LH, and estrogen in parents and offspring which indicates that the treatment with *F.* carica increases the proliferation of follicles which is also evident by histology and fertility data; since copulation occurred in less time, these results were repeated in F_1_ generation in a dose-dependent manner. The expression of SOD2, GPX1, CAT, and GR was increased in ovaries in the group treated with *F. carica* 100 mg/kg except for the expression of GPX 1 in F_0_ generation; this shows the antioxidant potential of the extract. *F. carica* increased prolactin levels in the female which indicates that it has the potential to increase milk production in lactating mothers.

The *F. carica* extract resulted in a highly significant increase in DHEAS levels and hence may be effective in the management of andropause in men. Andropause is a steady process nearly similar to menopause in women and occurs with a steady and persistent drop in testosterone and dehydroepiandrosterone production in men [23]. The *F. carica* extract may also be used in the management of menopausal women as it enhances the levels of estrogen and DHEAS. Both age and declining estradiol levels had significant detrimental effects on sexual functioning, libido, and sexual responsiveness in females. The drug is effective in dengue as it increases the levels of platelets. A hallmark of dengue infection is thrombocytopenia which is associated with abnormal platelet function [24].

## 5. Conclusion

*F. carica* is effective in enhancing conception and fertility in males and females by promoting ovulation and spermatogenesis. It also showed increased gene expression of antioxidant enzymes in both F_0_ and F_1_ generation producing an overall supportive effect in the reproduction process of both genders without any harmful effect on hematic, renal, and hepatic parameters. Results show that it may also be effective in andropause men and menopausal women. *F. carica* may also be effective in the management of dengue fever since has increased platelet and RBC count. However, the study has some constraints such as a lack of human studies which are essential for the general use of any drug in humans. Nonetheless, human studies may be performed prospectively to endorse the safety of *F. carica* extract in humans.

## Figures and Tables

**Table 1:** List of primer sequences used for RT-qPCR.

DNAid	Name	Forward sequence (5′⟶3′)	DNAid	Reverse sequence (5′⟶3′)
A210701-005D07	GAPDH	GCATCTTCTTGTGCAGTGCC	A210701-005B08	GATGGTGATGGGTTTCCCGT
A210701-005E07	SOD2	AGCTGCACCACAGCAAGCAC	A210701-005C08	TCCACCACCCTTAGGGCTCA
A210701-005F07	CAT	TCCGGGATCTTTTTAACGCCATTG	A210701-005D08	TCGAGCACGGTAGGGACAGTTCAC
A210701-005G07	GPX1	CGGTTTCCCGTGCAATCAGT	A210701-005E08	ACACCGGGGACCAAATGATG
A210701-005H07	GR	TGCACTTCCCGGTAGGAAAC	A210701-005F08	GATCGCAACTGGGGTGAGAA

GAPDH: Glyceraldehyde 3-phosphate dehydrogenase; SOD2: Manganese-dependent superoxide dismutase CAT: Catalase; GPX1: Glutathione peroxidase; GR: Glutathione reductase.

**Table 2:** Effect of the *F. carica* extract on blood biochemical changes in rats.

Factors	Control	FC 50 mg	FC 100 mg
AST (U/L)	20.5 ± 0.22	18.3 ± 0.39^*∗∗*^	18.1 ± 0.38^*∗∗*^
ALT (U/L)	20.1 ± 0.40	19.6 ± 0.37	18.9 ± 0.43
Total bilirubin (mg/dl)	0.86 ± 0.002	0.749 ± 0.01^*∗∗*^	0.779 ± 0.014^*∗∗*^
ALP (U/L)	111.6 ± 1.17	109.3 ± 0.95	112.3 ± 1.71
Troponin-T (ng/ml)	0.226 ± 0.003	0.215 ± 0.003	0.219 ± 0.002^*∗∗*^
Troponin-I (ng/ml)	0.087 ± 0.002	0.068 ± 0.001^*∗∗*^	0.047 ± 0.002^*∗∗*^
CKMB (IU/L)	17.8 ± 0.25	17.5 ± 0.22	17.5 ± 0.22
Creatinine (mg/dl)	0.845 ± 0.02	0.818 ± 0.01	0.847 ± 0.02
BUN (mmol/L)	9.15 ± 0.16	9.515 ± 0.12	9.327 ± 0.17

*n* = 10, data are shown as mean ± SEM. ^*∗∗*^*P* < 0.01 deliberated as highly significant and ^*∗∗*^*P* < 0.05 as significant.

**Table 3:** Effect of the *F. carica* extracts on hematological parameters in rats.

Parameters	Control	FC 50 mg	FC 100 mg
Hemoglobin (g/dl)	14.121 ± 0.05	13.984 ± 0.02	13.785 ± 0.19
RBC (x10^∧^12/L)	4.794 ± 0.20	5.388 ± 0.20	6.165 ± 0.10^*∗∗*^
WBC (x10^∧^9/L)	4.75 ± 0.14	5.14 ± 0.16	5.08 ± 0.12
Platelets (x10^∧^9/L)	358.8 ± 6.59	1042.1 ± 3.54^*∗∗*^	1017.5 ± 3.53^*∗∗*^

*n* = 10; data are shown as mean ± SEM. ^*∗∗*^*P* < 0.01 deliberated as highly significant and ^*∗*^*P* < 0.05 as significant.

**Table 4:** Effect on fertility indices of control and treated groups in F_0_ generation.

Extract doses (mg/kg)	Cohabitation period until copulation (days)	Fertility index (%) (No. Pregnant/no. Copulated)	Mean gestational length (days)	Mean no. of pups/litter	No. of pups expire till PND21	Mean weight at birth (g)	Male: Female ratio	Delivery index (%) (No. Delivered/no. Pregnant)	Mean weight at week 3 (g)
Control	27.3 ± 0.57	50 (3/6)	21 days	7.33 ± 0.57	Nil	4.56 ± 0.28	0.83 ± 0.14	100 (3/3)	22.12 ± 2.01
FC 50 mg	15 ± 0.81	66.6 (4/6)	20 days	8 ± 0.00	Nil	5.87 ± 0.22	1.00 ± 0.00	100 (4/4)	27.02 ± 2.43
FC 100 mg	3.2 ± 0.44	83.3 (5/6)	20 days	8 ± 0.00	Nil	6.08 ± 0.15	1.00 ± 0.00	100 (5/5)	32.32 ± 3.06

**Table 5:** Effect on fertility indices of control and treated groups in the F_1_ generation.

Extract doses (mg/kg)	Cohabitation period until copulation (days)	Fertility index (%) (No. Pregnant/no. Copulated)	Mean gestational length (days)	Mean no. of pups/litter	No. of pups expire till PND21	Mean weight at birth (g)	Male: Female ratio	Delivery index (%) (No. Delivered/no. Pregnant)	Mean weight at week 3 (g)
Control	32.50 ± 3.5	33.3 (2/6)	21 days	7.5 ± 0.7	Nil	4.57 ± 0.32	0.87 ± 0.18	100 (2/2)	24.07 ± 1.8
FC 50 mg	7.66 ± 0.58	50 (3/6)	20 days	8 ± 0.0	Nil	5.43 ± 0.22	1.00 ± 0.00	100 (3/3)	29.23 ± 2.7
FC 100 mg	1.6 ± 0.54	83.3 (5/6)	20 days	8 ± 0.0	Nil	6.2 ±0.33	1.00 ± 0.00	100 (5/5)	35.61 ± 4.1

**Table 6:** Hormonal comparison of male and female rats in the F_0_ generation.

Parameters	Control	FC 50 mg	FC 100 mg
GnRH male (lU/ml)	6.02 ± 0.08	6.17 ± 0.18	6.68 ± 0.17^*∗*^
GnRH female (lU/ml)	6.62 ± 0.82	6.62 ± 0.82	6.735 ± 0.81
FSH male (lU/ml)	0.43 ± 0.28	0.92 ± 0.11^*∗∗*^	1.80 ± 0.26^*∗∗*^
FSH female (lU/ml)	1.49 ± 0.30	5.09 ± 0.59^*∗∗*^	5.24 ± 0.57^*∗∗*^
LH male (lU/ml)	0.36 ± 0.06	0.62 ± 0.08^*∗∗*^	1.41 ± 0.28^*∗∗*^
LH female (lU/ml)	0.80 ± 0.009	3.18 ± 0.81^*∗*^	3.48 ± 0.77^*∗*^
Estradiol male (Pg/ml)	24.53 ± 3.20	35.38 ± 2.90^*∗∗*^	40.38 ± 2.81^*∗∗*^
Estradiol female (Pg/ml)	23.23 ± 2.68	48.3 ± 3.25^*∗∗*^	56.42 ± 3.09^*∗∗*^
Testosterone male (ng/ml)	0.83 ± 0.12	1.400 ± 0.27	1.45 ± 0.27
Testosterone female (ng/ml)	0.72 ± 0.008	0.95 ± 0.09	0.95 ± 0.07
DHT male (ng/dl)	18.00 ± 2.30	29.00 ± 2.65^*∗∗*^	35.00 ± 2.88^*∗∗*^
DHT female (ng/dl)	28.35 ± 3.29	29.15 ± 3.39	34.3 ± 2.76
DHEAS male (umol/l)	2.25 ± 0.29	7.01 ± 0.29^*∗∗*^	8.29 ± 0.27^*∗∗*^
DHEAS female (umol/l)	1.79 ± 0.26	5.65 ± 0.58	10.33 ± 2.88^*∗*^
Progesterone male (mg/ml)	12.42 ± 1.63	15.24 ± 1.56	13.09 ± 0.93
Progesterone female (mg/ml)	10.30 ± 1.47	10.73 ± 1.50	9.72 ± 1.16
Insulin male (IU/ml)	8.23 ± 0.49	13.11 ± 0.93^*∗*^	15.21 ± 1.38^*∗∗*^
Insulin female (IU/ml)	9.22 ± 1.33	9.34 ± 0.57	9.83 ± 0.57
Prolactin female ng/ml	7.38 ± 1.16	11.29 ± 1.32	25.59 ± 1.32^*∗∗*^

*n* = 6, data are shown as means ± SEM. ^*∗∗*^*P* < 0.01 deliberated as highly significant and ^*∗*^*P* < 0.05 as significant.

**Table 7:** Hormonal comparison of male and female rats in the F_1_ generation.

Parameters	Control	FC 50 mg	FC 100 mg
GnRH male (lU/ml)	6.29 ± 0.93	6.57 ± 0.81	7.38 ± 0.56
GnRH female (lU/ml)	7.64 ± 0.61	6.91 ± 0.82	7.06 ± 0.57
FSH male (lU/ml)	0.37 ± 0.04	0.92 ± 0.05^*∗∗*^	1.15 ± 0.22^*∗∗*^
FSH female (lU/ml)	1.18 ± 0.24	1.93 ± 0.28	3.97 ± 0.29^*∗∗*^
LH male (lU/ml)	0.32 ± 0.029	0.72 ± 0.06^*∗∗*^	0.68 ± 0.08^*∗∗*^
LH female (lU/ml)	1.14 ± 0.14	1.64 ± 0.29	1.82 ± 0.28
Estradiol male (Pg/ml)	19.17 ± 2.55	23.67 ± 2.80	25.83 ± 3.016
Estradiol female (Pg/ml)	18.06 ± 1.17	44.85 ± 1.45^*∗∗*^	50.05 ± 2.89^*∗∗*^
Testosterone male (ng/ml)	1.17 ± 0.13	1.04 ± 0.14	1.34 ± 0.13
Testosterone female (ng/ml)	0.66 ± 0.01	0.86 ± 0.19	0.99 ± 0.19
DHT male (ng/dl)	18.33 ± 1.28	21.33 ± 1.60	24.5 ± 2.92
DHT female (ng/dl)	27.84 ± 1.17	28.84 ± 1.08	29.84 ± 1.08
DHEAS male (umol/l)	2.9 ± 0.28	6.81 ± 0.28^*∗∗*^	6.99 ± 0.28^*∗∗*^
DHEAS female (umol/l)	1.04 ± 0.03	5.63 ± 0.80^*∗∗*^	7.34 ± 0.89^*∗∗*^
Progesterone male (mg/ml)	11.45 ± 0.59	11.83 ± 0.54	12.02 ± 0.58
Progesterone female (mg/ml)	8.22 ± 1.07	8.79 ± 0.01	8.32 ± 1.07
Insulin male (IU/ml)	8.25 ± 0.67	9.11 ± 0.57	9.47 ± 0.68
Insulin female (IU/ml)	8.31 ± 0.47	8.59 ± 0.28	8.59 ± 0.62
Prolactin female ng/ml	6.52 ± 0.82	6.47 ± 0.82	6.02 ± 0.58

*n* = 6, data are shown as means ± SEM. ^*∗∗*^*P* < 0.01 deliberated as highly significant and ^*∗*^*P* < 0.05 as significant.

**Table 8:** Description of female rat ovaries in control and treated F_0_ and F_1_ generation.

Variable	F_0_ Control	F_0_ 50 mg	F_0_ 100 mg	F_1_ Control	F_1_ 50 mg	F_1_ 100 mg
Number of pre primordial follicles	2.5 ± 0.55	2.66 ± 0.51	2.83 ± 0.41	2.3 ± 0.82	1.83 ± 0.41	2.5 ± 0.55
Number of primary follicles	1.33 ± 0.52	1.66 ± 0.52	1.83 ± 0.41	1.33 ± 0.52	1.33 ± 0.52	1.5 ± 0.55
Number of secondary follicles	1.33 ± 0.52	1.33 ± 0.52	1.17 ± 0.41	1.5 ± 0.54	1.33 ± 0.52	1.66 ± 0.52
Number of antral follicles	0.5 ± 0.55	0	0	0	0	0.6±0.52
Number of preovulatory griffin follicles	0	0	0.33 ± 0.52	0	0	0.83 ± 0.41
Number of corpora lutea	3.33 ± 0.52	1.5 ± 0.55	1.5 ± 0.55	1.5 ± 0.55	1.33 ± 0.52	1.5 ± 0.55

*n* = 6.

## Data Availability

The data used to support the findings of this study are available from the corresponding author on request.

## Additional Points

The findings of the present study lead us toward the resolution of an important issue of infertility affecting a large number of people. This study seems to provide clues for the resolution of this serious issue in either sex without any side effects. Thus, the outcomes of this study provide access to a relatively safer procedure to deal with this serious problem. The results also help in developing the right approach for the use of natural products by providing physicians with scientific grounds for their use. Therefore, the results of this study will help to alter the field of pharmacology in the treatment of disorders through the use of natural medicines with negligible adverse effects.
